# Erythrocytic α-Synuclein as a potential biomarker for Parkinson’s disease

**DOI:** 10.1186/s40035-019-0155-y

**Published:** 2019-05-15

**Authors:** Chen Tian, Genliang Liu, Liyan Gao, David Soltys, Catherine Pan, Tessandra Stewart, Min Shi, Zhiying Xie, Na Liu, Tao Feng, Jing Zhang

**Affiliations:** 1 Department of Pathology, Peking University School of Basic Medical Sciences, Peking University, Beijing, China; 2 Department of Pathology, Peking University Third Hospital, Peking University, Beijing, China; 3 Center for Neurodegenerative Disease, Department of Neurology, Beijing Tiantan Hospital, Capital Medical University, Beijing, China; 4 China National Clinical Research Center for Neurological Diseases, Beijing, China; 5 Parkinson’s Disease Center, Beijing Institute for Brain Disorders, Capital Medical University, Beijing, China; 6 Department of Pathology, University of Washington School of Medicine, Seattle, WA USA; 7 Beijing Key Laboratory of Research and Transformation on Neurodegenerative Diseases Biomarkers, Beijing, China; 8 Department of neurology, Qingdao Municipal Hospital, Qingdao University, Qingdao, China; 9 Department of Neurology, Peking University Third Hospital, Peking University, Beijing, China

**Keywords:** Parkinson’s disease, α-Synuclein, Erythrocyte, Electrochemiluminescence

## Abstract

**Background:**

Erythrocytes are a major source of peripheral α-synuclein (α-Syn). The goal of the current investigation is to evaluate erythrocytic total, oligomeric/aggregated, and phosphorylated α-Syn species as biomarkers of Parkinson’s disease (PD). PD and healthy control blood samples were collected along with extensive clinical history to determine whether total, phosphorylated, or aggregated α-Syn derived from erythrocytes (the major source of blood α-Syn) are more promising and consistent biomarkers for PD than are free α-Syn species in serum or plasma.

**Methods:**

Using newly developed electrochemiluminescence assays, concentrations of erythrocytic total, aggregated and phosphorylated at Ser129 (pS129) α-Syn, separated into membrane and cytosolic components, were measured in 225 PD patients and 133 healthy controls and analyzed with extensive clinical measures.

**Results:**

The total and aggregated α-Syn levels were significantly higher in the membrane fraction of PD patients compared to healthy controls, but without alterations in the cytosolic component. The pS129 level was remarkably higher in PD subjects than in controls in the cytosolic fraction, and to a lesser extent, higher in the membrane fraction. Combining age, erythrocytic membrane aggregated α-Syn, and cytosolic pS129 levels, a model generated by using logistic regression analysis was able to discriminate patients with PD from neurologically normal controls, with a sensitivity and a specificity of 72 and 68%, respectively.

**Conclusions:**

These results suggest that total, aggregated and phosphorylated α-Syn levels are altered in PD erythrocytes and peripheral erythrocytic α-Syn is a potential PD biomarker that needs further validation.

**Electronic supplementary material:**

The online version of this article (10.1186/s40035-019-0155-y) contains supplementary material, which is available to authorized users.

## Background

Parkinson’s disease (PD) is a common age-related movement disorder. Currently, clinical diagnosis of PD mainly relies on motor symptoms such as resting tremor, bradykinesia, muscle rigidity and balance disorders [1]. Previous studies have examined α-synuclein (α-Syn), a key protein critically involved in PD pathogenesis, as a potential biomarker. Most studies focus on cerebrospinal fluid (CSF) [2–4], which is in direct contact with the brain and spinal cord. However, obtaining CSF routinely at typical clinics is challenging, due to the invasive nature of the procedure and need for highly skilled staff to perform it. Additionally, the performance of CSF α-Syn in PD diagnosis has been shown to be only low to moderate [5, 6].

Because collection of peripheral blood samples is considerably easier, defining biomarkers in blood has numerous advantages. However, assessment of plasma/serum α-Syn levels has not yielded consistent results [7–12] partially because > 99% of blood α-Syn is located in erythrocytes [13], and hemolysis, in vivo or in vitro, markedly affects α-Syn values [14]. Further, the interaction of α-Syn with lipid membranes is implicated in its physiological and pathological roles [15–17], and PD patients exhibit morphological abnormalities of erythrocytes [18], possibly via the known effects of aggregated α-Syn on cell membranes [19, 20]. These factors suggest that expression and function of α-Syn forms may differ between erythrocyte compartments, with each representing separate aspects of α-Syn pathology. Moreover, plasma oligomeric [21] and phosphorylated [22] α-Syn, two species associated with its mechanisms of toxicity [23–28], and α-Syn oligomers in erythrocytes [29, 30], have also been measured (though not in separate cellular components) with encouraging results. However, these preliminary studies by us and others tested small sample cohorts with less robust immunoassays, and thus further independent validation studies are needed.

The present study is designed to test the hypotheses that total, aggregated (including oligomers and larger, soluble aggregates) and/or phosphorylated α-Syn in membrane and cytosolic fractions derived from peripheral erythrocytes are altered in PD and could serve as biomarker candidates for either disease diagnosis or disease severity correlation. In this study, we have used a relatively large clinical cohort and developed more robust immunoassays to validate the potential of erythrocytic α-Syn as a PD biomarker. Our study examined α-Syn separately in the membrane and cytosolic fractions of the erythrocyte. The expression and function of α-Syn forms may differ between erythrocyte compartments, with each representing separate aspects of α-Syn pathology.

## Materials and methods

### Participants

Standard protocol approvals, registrations, and patient consents: The study protocol was approved by the Institutional Review Boards of Peking University, Peking University Third Hospital, Beijing, China. Written consents were obtained from all subjects.

The study cohort included 225 patients diagnosed with idiopathic PD, and 133 healthy control subjects recruited from Capital Medical University, Tiantan Hospital, Beijing, China. All PD patients met diagnostic criteria in accordance with those of the United Kingdom PD Society Brain Bank [31], and were treated with medication. All control participants were recruited from the physical examination center in TianTan Hospital, and were healthy subjects without history of any neurological disorders. Subjects underwent evaluation including medical history, and assessment of motor and cognitive functions. Any control subjects or PD patients with hematological diseases or inflammatory diseases were excluded from this study. The characteristics of the cohort are presented in Table 1, and are quite similar to those in our previous studies [32, 33], including distribution of Unified Parkinson disease rating scale (UPDRS; part III) [34], a typical measure of movement dysfunction, and Montreal Cognitive Assessment (MoCA), a screening instrument for the detection of cognitive impairment or dementia in PD [35]. No subjects in the present study were included in our pilot study [29], which used a traditional ELISA and a smaller cohort to measure α-Syn oligomers in erythrocytes.

**Table 1:** Summary of participant demographic and clinical information

	PD	Control
Cases	225	133
Age (year)^a^	61.01 ± 10.68	60.0 ± 9.10
Sex (Male:Female)	118:107	68:65
UPDRS III^a^	20.95 ± 11.44	–
Disease duration (year)^a^	6.28 ± 5.00	–
MoCA^a^	21.76 ± 5.12	–

^a^ Data shown are mean ± S. D. *MoCA* Montreal cognitive assessment, *PD* Parkinson’s disease, *UPDRS III* Unified Parkinson disease rating scale, part III

### Erythrocyte collection and separation

Whole blood (5 ml) was collected in EDTA-coated tubes and aliquoted. The blood was centrifuged at 1500×*g* and 4 °C for 10 min, and plasma and leukocytes were removed. Pelleted erythrocytes were washed three times in PBS and centrifuged at 1500×*g* for 10 min. The supernatant was removed and the pellets were aliquoted and stored at − 80 °C within 90 min of blood collection. Samples were thawed only at the time of analysis.

To separate the cytosolic and membrane fractions, erythrocytes were subjected to two sequential freeze (− 80 °C) and thaw (room temperature) cycles, then centrifuged at 14000×*g* and 4 °C for 10 min. The cytosolic protein-containing supernatant (cytosolic fraction) was removed and stored at − 80 °C, while the membrane pellet was subsequently washed 3 times with PBS and centrifuged at 14000×*g* and 4 °C for 10 min. The membrane pellet was solubilized with STET lysis buffer (0.1 mmol/L NaCl, 10 mmol/L Tris pH 8.0, 1 mmol/L EDTA, 1% Triton 100), incubated on ice for 30 min, and centrifuged at 14000×g and 4 °C for 10 min to pellet any remaining insoluble material. The membrane protein-containing supernatant (membrane fraction) was isolated and stored at − 80 °C. The quality of the separation was assessed by probing specific membrane (glycophorin A [CD235a]) and cytoplasmic (glyceraldehyde-3-phosphate dehydrogenase [GAPDH]) proteins by western blot.

Protein concentrations in erythrocyte membrane and cytosol fractions were measured using the bicinchoninic acid (BCA) protein assay kit (Pierce/Thermo Fisher Scientific, Rockford, IL, USA) at an absorbance of 562 nm relative to a protein standard.

### Electrochemiluminescence (ECL) immunoassays for total, aggregated, and phosphorylated α-Syn quantification

Standard proteins were recombinant unphosphorylated (Alpha-synuclein Protein – monomer; Cat# PR-001, Proteos, Inc., Kalamazoo, MI, USA) or phosphorylated (Alpha-synuclein Protein – Phospho S129; Cat#PR-004, Proteos, Inc.) monomers, or filaments (Alpha-synuclein Protein – filament; Cat# PR-002, Proteos, Inc.). Phosphorylated standards were semisynthetic full length proteins generated by ligation of a recombinant peptide to a synthetic phosphopeptide. Filaments were generated by the manufacturer from purified monomers, and the concentration was assessed by BCA protein assay. Filaments were reconstituted in distilled, deionized water at a concentration of 1 mg/ml and frozen at − 80 °C before use. Immediately before the assay was run, the calibrators were diluted in Diluent 35 (MSD, Rockville, MD, USA) to 1 μg/ml and sonicated for 1 min before preparation of the standard curve by serial dilution.

Anti-α-Syn clone 42 (624,096, BD Bioscience, San Jose, CA, USA) was labelled with Sulfo-TAGs according to MSD’s instructions and used as the detector for all three assays. Anti-α-Syn MJFR-1 clone 12.1 (ab138501, Abcam, Cambridge, MA, USA), conformation specific, anti-α-Syn filaments MJFR-14 (ab209538, Abcam), and anti-phosphorylated α-Syn at Ser129 (pS129; BioLegend, San Diego, CA, USA) antibodies were biotinylated and coated onto standard 96-well Meso Scale Discovery (MSD) U-Plex plates by incubating the plates with 1 μg/ml capture antibody solutions for 2 h at room temperature with 600 rpm shaking, according to the manufacturer’s instructions. After washing three times with 150 μl wash buffer (MSD), plates were blocked with 150 μL Diluent 35 (MSD) for 1 h while shaking at 600 rpm at room temperature, then washed three times in wash buffer. Samples were diluted (cytosol samples were diluted 1:10^5^ and membrane samples were diluted 1:10^4^ for the total α-Syn assay; both cytosol and membrane samples were diluted 1:100 for the aggregated α-Syn assay; cytosol samples were diluted 1:25 and membrane samples were diluted 1:15 for the pS129 assay; all in Diluent 35) and incubated with recombinant α-Syn standards for 1 h at room temperature and while shaking at 600 rpm. After washing three times, Sulfo-TAG-labelled anti-α-Syn clone 42 antibody (1μg/ml) was added and incubated for 1 h at room temperature with 600 rpm shaking. After washing three times, 150 μL of 2× Read Buffer T (MSD) was applied to each well and plates were analyzed in a Sector Imager 6000 (MSD). Data analysis was performed with the MSD Discovery Workbench 3.0 Data Analysis Toolbox.

Spike–in recovery was performed to test the assay accuracy by spiking various concentrations of the corresponding standard proteins for each assay into the sample matrix and calculating the recovery as follows: (observed spiked sample concentration – observed unspiked sample concentration) / expected added concentration * 100%. For the total α-Syn assay, 100, 200, and 400 pg/ml of unphosphorylated α-Syn monomers were spiked into the cytosolic or membrane erythrocyte matrix. For the aggregated α-Syn assay, 250, 500, and 1000 pg/ml of α-Syn filaments were used for the cytosolic erythrocyte matrix, and 50, 100, and 200 pg/ml of α-Syn filaments were used for the membrane erythrocyte matrix. For the pS129 assay, 250, 500, and 1000 pg/ml of α-Syn Phospho S129 were used for the cytosolic erythrocyte matrix, and 50, 100, and 200 pg/ml of α-Syn Phospho S129 were used for the membrane erythrocyte matrix.

Additionally, the assay accuracy was tested by linearity-of-dilution for each assay. The concentrations of total α-Syn in the cytosolic fraction were measured at dilution ratios of 1:10^3^, 1:10^4^, 1:10^5^ and 1:10^6^ in Diluent 35 and in the membrane fraction it was tested at dilution ratios of 1:10^3^, 1:10^4^, and 1:10^5^. The concentrations of cytosolic pS129 were measured at dilution ratios of 1:25, 1:50 and 1:100 in Diluent 35 and the concentrations of membrane pS129 were measured at dilution ratios of 1:7.5, 1:15 and 1:30. The concentrations of aggregated α-Syn in cytosol were tested at dilution ratios of 1:1000, 1:100 and 1:10 in Diluent 35 and in the membrane fraction it was tested at dilution ratios of 1:1000, 1:100 and 1:10.

### Antibody/assay specificity tests

Aβ_42_ oligomers as a negative control for the aggregated α-Syn assay were prepared according to a published protocol [36]. Briefly, 2 μl of 5 mM Aβ_42_ monomers (62–0-80, American Peptide, Sunnyvale, CA, USA) dissolved in DMSO were diluted to 100 μM by adding 98 μl of ice cold 4 mM HEPES (pH 8.0). The sample was sonicated in a water bath for 10 min before incubating for 24 h at 4 °C. Soluble Aβ_42_ oligomers/aggregates were obtained after sonication and centrifugation and stored at − 80 °C before use. In the assay, Aβ_42_ oligomers were captured by using the conformation specific, anti-α-Syn filaments MJFR-14 and detected by using either the Sulfo-TAG-labelled Anti-α-Syn clone 42 or a Sulfo-TAG-labelled Aβ antibody MOAB-2 (NBP2–13075, Novus Biologicals, Centennial, CO, USA).

Immunodepletion was also performed to test the antibody/assay specificity using antibody-coupled superparamagnetic microbeads following a protocol adapted from our pilot study [35]. Briefly, 10 μg of anti-α-Syn antibody (MJFR-1, ab138501, Abcam) or anti-aggregated α-Syn antibody (MJFR-14, ab209538, Abcam) were coated to one set (1 mg) of M-270 beads using a Dynabeads Antibody Coupling Kit (Invitrogen/Thermo Fisher Scientific, Carlsbad, CA, USA). 50 μl of erythrocyte lysates were diluted 1:20 with PBS (pH 7.4). One set of antibody-coated beads and 900 μl of diluted erythrocyte lysates were incubated for 24 h at 4 °C with gentle rotation. Immunodepleted supernatants were measured for α-Syn species in immunoassays.

### Statistical analysis

Total α-Syn was normalized to total erythrocytic protein levels in the same subcellular compartment, and concentrations of aggregated and pS129 α-Syn were normalized to total α-Syn levels before analysis. Both raw and normalized values are reported. Because the biomarker data produced a skewed distribution that was not remedied by transformation of the variables, non-parametric Mann Whitney U test was used to compare group means and Spearman’s rank correlation coefficient (ρ) was used to analyze correlation between biomarkers and PD severity or between different α-Syn species within each cellular compartment. *P < 0.05* was considered significant*.* To generate a multivariable logistic regression model suitable to analyze independent influencing factors for PD diagnosis, binary logistic regression was performed using the Backward LR (likelihood ratio) method. The area under the receiver operating characteristic (ROC) curve was analyzed to determine the most appropriate cutoff values for PD and control groups. Analyses were performed using SPSS 23.0 software (SPSS Inc., Chicago, IL, USA) and GraphPad Prism 6 (GraphPad Software, La Jolla California USA).

## Results

### Establishment of the ECL assays

To reliably quantify total, aggregated and pS129 levels in human erythrocytes, specific and sensitive novel ECL methods were developed on the MSD platform.

**Fig. 1:**
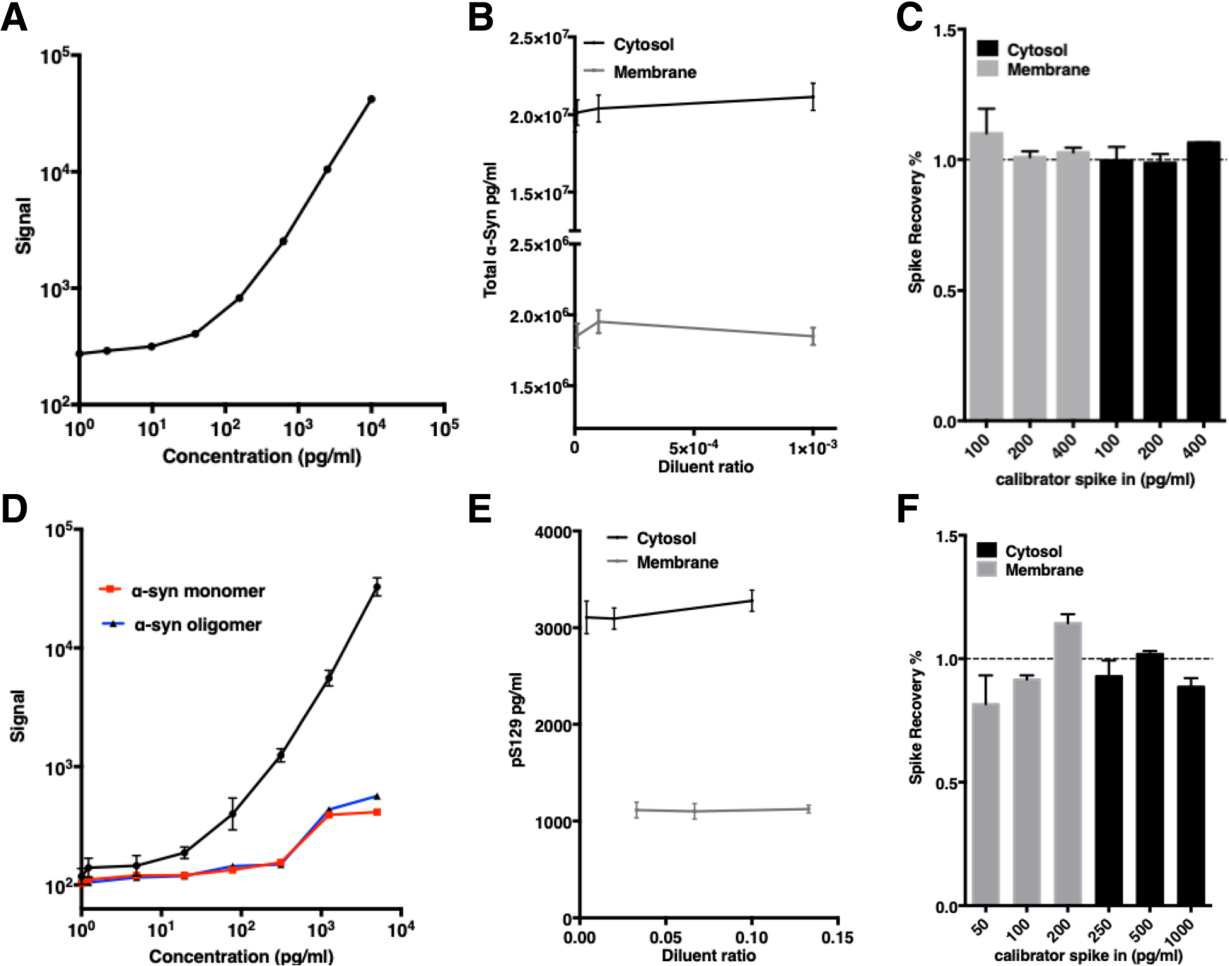
Establishment and characterization of the total α-Syn and pS129 ECL assay systems. **a** A representative standard curve of the the total α-Syn assay. The detection range was from 5 pg/ml to 10 ng/ml (R^2^ = 0.999). **b** Accuracy of the total α-Syn assay was tested by using Linearity-of-dilution, with dilutions of 1:10^3^, 1:10^4^, 1:10^5^ and 1:10^6^ in Diluent 35 in the cytosol and 1:10^3^, 1:10^4^, and 1:10^5^ in the membrane fraction. **c** Total α-Syn assay accuracy was also tested by spike–in recovery using 100, 200, and 400 pg/ml of unphosphorylated α-Syn monomers. **d** A representative standard curve of the pS129 assay (Black line, R^2^ = 0.999). The detection range was from 10 pg/ml to 5 ng/ml. Assay specificity was measured by detecting unphosphorylated α-Syn monomers (red line), or unphosphorylated α-Syn aggregates (blue line) at the same concentrations. **e** Linearity-of-dilution of the pS129 assay was assessed using dilutions of 1:25, 1:50 and 1:100 in the cytosol and 1:7.5, 1:15 and 1:30 in the membrane fraction. **f** PS129 assay spike–in recovery was tested by spiking in 250, 500, and 1000 pg/ml of the pS129 standard in the cytosolic fraction and 50, 100, and 200 pg/ml of the standard in the membrane fraction

The total α-Syn ECL assay has a broad detection range from 5 pg/ml to 10 ng/ml (Fig. 1a). The day-to-day and plate-to-plate signal variability were low (CVs < 10% within the 16 plates analyzed). Assay accuracy was measured by linearity-of-dilution, and by spiking human recombinant α-Syn monomer into erythrocytic cytosolic and membrane protein fractions. The recoveries of the linearity-of-dilution tests were 104.9 ± 0.3% and 102.6 ± 0.6% (Fig. 1b), and spike-in recoveries were 101.7 ± 2.2% and 104 ± 3.2%, for monomer cytosol and membrane fractions, respectively (Fig. 1c).

The phosphorylated α-Syn assay capture antibody targets pS129 α-Syn. The detection range is 10 pg/ml to 5 ng/ml (Fig. 1d), with low day-to-day and plate-to-plate signal variability (CVs < 10%). The recoveries of linearity-of-dilution for cytosolic and membrane fractions were 102.9 ± 1.3% and 100.0 ± 0.5%, respectively (Fig. 1e), and the spike-in recoveries were 94.5 ± 2.9% and 95.9 ± 6.0%, for cytosolic and membrane fractions, respectively (Fig. 1f). The specificity of the assay was tested by measuring recombinant standard unphosphorylated monomeric and oligomeric α-Syn. Very little signal was detected (e.g., < 2% of unphosphorylated monomers compared to pS129 at 10 ng/ml), indicating low reactivity of the pS129 antibody with unphosphorylated species (Fig. 1d).

**Fig. 2:**
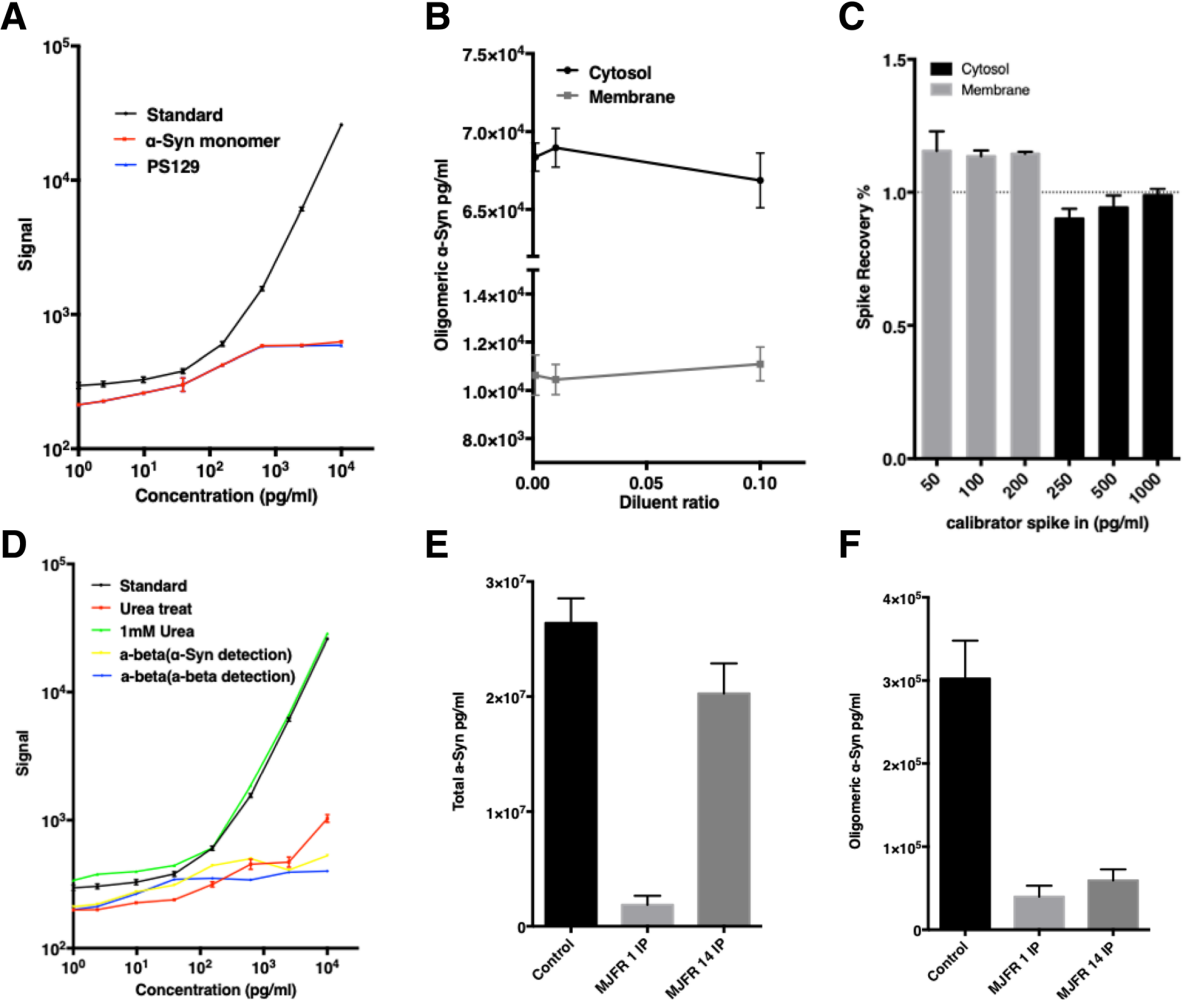
Establishment and characterization of the aggregated α-Syn ECL assay system. **a** The aggregated α-Syn standard curve was generated over a range of 9 pg/ml to 10 ng/ml (black line; R^2^ = 0.999). Specificity was tested by measuring unphosphorylated (red line) or phosphorylated (blue line) monomeric species run in the same aggregated α-Syn assay. **b** Linearity-of-dilution of the aggregated α-Syn assay was assessed by using dilutions of 1:1000, 1:100 and 1:10 in the cytosolic and membrane fractions. **c** Spike–in recovery of the aggregated α-Syn assay was tested by spiking 250, 500, and 1000 pg/ml of α-Syn aggregates into the cytosolic fraction or 50, 100, and 200 pg/ml into the membrane fraction. **d** Specificity of MJFR14 conformational specific antibody was examined. Red line: aggregated α-Syn signals after dissociation using 8 M Urea treatment. Green line: aggregated α-Syn standard curves incubated with 1 mM Urea, the same final concentration as included in the disaggregated calibrator assay. Yellow line: Aβ oligomers detected using the aggregated α-Syn assay (MJFR14 antibody and anti-α-Syn detection antibody). Blue line: Aβ oligomers captured with the conformation-specific α-Syn antibody and an Aβ-specific detection antibody. **e** The total α-Syn concentrations (measured by using the total α-Syn assay) before and after immunoprecipitation using MJFR 1(recognizing “total” α-Syn, including monomeric and oligomeric/aggregated forms) or MJFR 14 (recognizing aggregated α-Syn only) in erythrocyte samples. **f** The aggregated α-Syn concentrations (measured by using the aggregated α-Syn assay) before and after immunoprecipitation using MJFR 1or MJFR 14 in erythrocyte samples

The aggregated α-Syn assay utilizes an antibody that recognizes the conformational changes undergone by α-Syn upon aggregation. Both oligomers and larger soluble aggregates, including those derived from sonicated fibrils, together encompassed by the general term “aggregates” are recognized by the assay. The aggregated α-Syn assay has a detection range from 9 pg/ml to 10 ng/ml (Fig. 2a), and a low day-to-day and plate-to-plate signal variability (CVs < 10%). The recoveries of linearity-of-dilution for cytosolic and membrane fractions were 99.3 ± 0.4% and 101.8 ± 1.7%, respectively (Fig. 2b), and the spike-in recoveries for erythrocytic cytosolic and membrane samples were 94.5 ± 2.2% and 114 ± 2.3%, respectively.

The specificity of the developed assay for aggregated α-Syn was tested by comparing the signal detected for monomeric α-Syn to soluble aggregate calibrator; very little signal was detected (e.g., < 2% of monomers compared to soluble aggregates at 10 ng/ml), suggesting low affinity of the assay for monomeric α-Syn species (Fig. 1d). When the aggregated calibrator was denatured by pre-treatment with 8 M urea for 3 h, followed by dilution, the oligomer-specific signal was eliminated. Addition of the same final concentration of urea (1 mM) in the assay had no effect on oligomer signal (Fig. 2d). To further demonstrate the specificity of the assay, we performed an experiment using the conformation-specific α-Syn antibody to capture oligomeric Aβ, followed by detection using either the same anti-α-Syn antibody used in the assay (to demonstrate the specificity of the overall assay), or an alternative detection antibody against Aβ (to demonstrate the specificity of the conformation-specific capture antibody in particular). The low resulting signal (1.5 and 2% of the signal using an equivalent concentration of aggregated α-Syn, respectively) further indicates the specificity for aggregated α-Syn (Fig. 2d). Additionally, we found that immunodepletion of either total (Fig. 2e) or aggregated (Fig. 2f) α-Syn, followed by measurement of the sample using the aggregated or total α-Syn assay, resulted in greatly reduced signal of aggregated α-Syn.

### Characterization of erythrocyte membrane and cytoplasmic component properties

To determine the quality of the separation of erythrocyte membrane and cytoplasmic components, western blots were performed using antibodies against CD235a (a sialoglycoprotein present on erythrocyte membrane) and GAPDH (expressed in erythrocytic cytoplasm). CD235a was detectable only on the membrane fraction and GAPDH was present only in the cytoplasmic fraction, indicating good separation (Additional file 1: Figure S1).

### The relationship between erythrocytic α-Syn and PD diagnosis and severity

We compared different α-Syn species by fraction in PD and control (raw and normalized values are reported in Table 2). Of note, erythrocyte content of blood samples varied substantially by subject. The concentrations of total α-Syn were normalized to total erythrocytic protein. In order to better separate changes in specific forms from changes in total α-Syn, the concentrations of aggregated and pS129 α-Syn were normalized to total α-Syn.

**Table 2:** Erythrocytic levels of total, phosphorylated, and aggregated α-Syn in patients with PD and healthy controls

Analyte	Fraction	Raw (±SEM) pg/ml			Normalized(±SEM) pg/μg		
		PD	Control	P	PD	Control	P
Total α-Syn^a^	Cytosol	33,942,290.92 ± 56,783.75	34,160,888.80 ± 102,781.46	0.45	76.44 ± 0.08	85.34 ± 0.26	0.203
	Membrane	3,443,923.88 ± 8521.66	3,169,731.45 ± 15,787.25	0.005	108.60 ± 0.26	94.71 ± 0.26	0.008
Aggregated α-Syn^b^	Cytosol	126,184.16 ± 210.16	131,977.36 ± 502.61	0.10	4024.52 ± 7.86	4316.47 ± 20.66	0.47
	Membrane	85,028.97 ± 184.99	56,531.94 ± 190.85	< 0.0005	27,284.05 ± 44.17	21,098.82 ± 75.82	<0.0005
PS129^b^	Cytosol	18,223.73 ± 155.50	2028.41 ± 13.10	< 0.0005	636.05 ± 6.03	67.36 ± 0.48	<0.0005
	Membrane	891.03 ± 2.23	584.16 ± 3.87	< 0.0005	315.35 ± 0.95	255.05 ± 1.98	<0.0005

^a^ Total α-Syn concentrations were normalized to the total protein levels in the same erythrocytic subcellular compartment, and are expressed in unit of pg (total α-Syn)/μg (total protein)

^b^ Oligomeric or phospho (pS129) α-Syn concentrations were normalized to the corresponding total α-Syn levels, and are expressed in units of pg (oligo α-Syn)/μg (total α-Syn) and pg (pS129)/μg (total α-Syn), respectively

*PD* Parkinson’s disease, *SEM* standard error of the mean, *α-Syn* α-synuclein

**Fig. 3:**
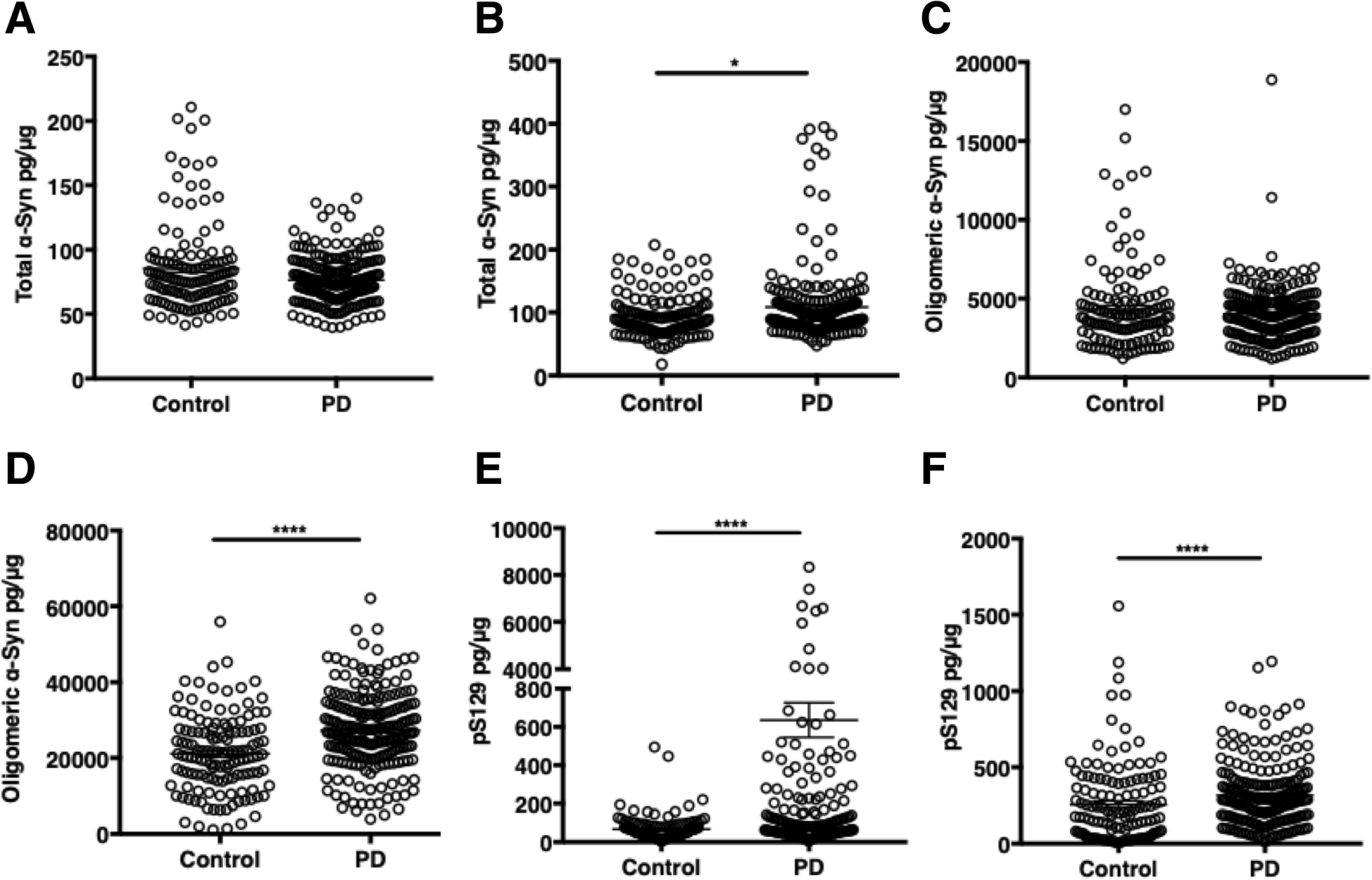
The erythrocytic levels of α-Syn species in patients with Parkinson’ disease (PD) and healthy controls. **a** Cytosolic total α-Syn, normalized to cytosolic total proteins (pg/μg); (**b**) Membrane total α-Syn, normalized to membrane total proteins (pg/μg); *, *p* = 0.008 ((Mann Whitney U test); (**c**) Cytosolic aggregated α-Syn, normalized to cytosolic total α-Syn (pg/μg); (**d**) Membrane aggregated α-Syn, normalized to membrane total α-Syn (pg/μg); ****, *p* < 0.0005; (**e**) Cytosolic pS129, normalized to cytosolic total α-Syn (pg/μg); ****, *p* < 0.0005; (**f**) Membrane pS129, normalized to membrane total α-Syn (pg/μg); ****: *p* < 0.0005

In this cohort, total α-Syn was significantly higher in the membrane fraction of PD subjects compared to controls (*p* = 0.008; Fig. 3b, Table 2), but trended lower in the cytosolic fraction (*p* = 0.203; Fig. 3a, Table 2). There was no significant difference in erythrocytic aggregated α-Syn in the cytosolic fraction (*p* = 0.469; Fig. 3c, Table 2), but in the membrane fraction, it was significantly higher in PD than in control (*p <* 0.0005; Fig. 3d, Table 2). pS129 was higher in PD than in control, particularly in the cytosolic fraction (*p <* 0.0005; Fig. 3e, Table 2).

Spearman’s rank correlation coefficient (ρ) was applied to analyze the correlation between different α-Syn species within each cellular compartment. We found that there was a weak, but significant, correlation between aggregated and total α-Syn in the cytosol fraction (ρ = 0.16, *p* = 0.02; Additional file 1: Figure S3A), but pS129 and total or aggregated α-Syn was not associated (*p* > 0.05; Additional file 1: Figure S3B, S3C). In the membrane fraction, aggregated α-Syn was also correlated with total α-Syn (ρ = 0.50, *p* < 0.01; Additional file 1: Figure S3D) and pS129 (ρ=0.33, p<0.01; Additional file 1: Figure S3F). There was no significant correlation between total α-Syn and pS129 in erythrocyte membrane (*p* > 0.05; Additional file 1: Figure S3E).

**Fig. 4:**
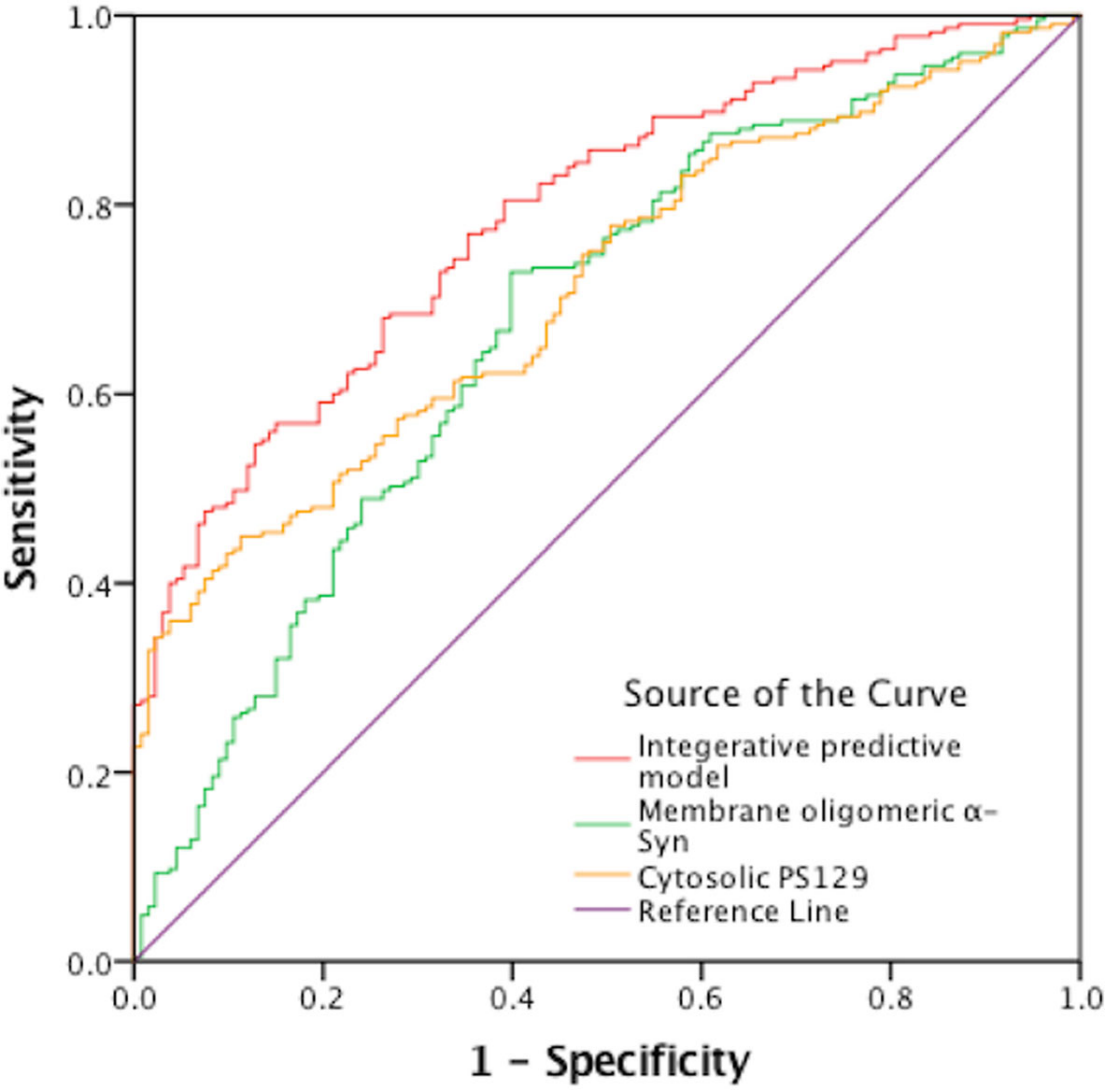
The receiver operating characteristic curves for erythrocytic α-Syn species and the integrative model. Green line: erythrocytic membrane aggregated α-Syn (AUC = 0.67); Yellow line: erythrocytic cytosolic pS129 (AUC = 0.71); Red line: the integrative model including membrane aggregated α-Syn, cytosolic pS129, and age (AUC = 0.79)

To evaluate the diagnostic utility of erythrocyte α-Syn, a ROC analysis was performed based on each analyte independently. The areas under the curve (AUCs) of the individual analytes with the best separation between groups were 0.67 and 0.71 for erythrocytic membrane aggregated α-Syn and cytosolic pS129, respectively (Fig. 4). We also assessed the performance of the oligomeric/aggregated α-Syn alone in whole erythrocytes (normalized to erythrocyte total proteins), as in our previous pilot study [29], but the results (AUC = 0.76) were not confirmed in this larger, independent cohort with more robust ECL assays (AUC = 0.61; Additional file 1: Figure S2).

Next, a step-wise logistic analysis was performed to select the best predictors, including the erythrocyte α-Syn forms that differed most between PD and healthy controls, along with age and gender. Membrane aggregated α-Syn, cytosolic pS129, and age were algorithmically selected as the major influencing factors for PD diagnosis (Table 3), and included in the integrated model. Based on the ROC analysis, the model was able to discriminate PD from control with an AUC of 0.79 (95% CI 0.74–0.84). Sensitivity was 72% and specificity was 68% in this cohort with a cutoff of 0.58 (Fig. 4).

**Table 3:** Logistic regression analysis

Variables	β	*P* value	95% CI
Membrane oligomeric α-Syn (pg/μg)	0.051	< 0.0001	1.025~1.080
Cytosolic pS129 (pg/μg)	0.006	0.001	1.002~1.0079
Age (year)	0.057	< 0.001	1.027~1.091
Constant	−4.942		

We also collected clinical data including UPDRS III (motor) score, disease duration and MoCA score to reflect the severity of different aspects of PD. We found no significant correlations between erythrocyte α-Syn species and any of the three clinical measures except that erythrocytic membrane pS129 was negatively correlated with MoCA (Spearman Correlation Coefficient *ρ* = − 0.17, *p* = 0.011), though the clinical relevance of this observation needs to be further investigated.

## Discussion

### Development of highly sensitive and accurate ECL assays to quantify erythrocyte α-Syn in peripheral blood

We established highly sensitive, accurate ECL assays to measure erythrocytic total, aggregated and pS129 α-Syn, with low intra- and inter-run variations, achieving sensitivity comparable to those using the Luminex platform [32, 33] and much higher than traditional [37] or even recently improved [38] ELISA for total and pS129 α-Syn. We applied these assays to a large cohort with comprehensive clinical information collected, in order to assess their function as PD biomarkers in this study. Notably, the aggregated α-Syn assay uses a recently developed antibody that recognizes the conformation taken by α-Syn in oligomers and aggregates [39]. Thus, the specificity of the capture antibody is vital for interpreting the results of the assay. We performed several experiments to demonstrate the specificity of the antibody and assay (recognition of phosphorylated or unphosphorylated monomeric α-Syn, disrupting aggregated standards, immunodepletion, and detection of oligomeric Aβ). Together, these studies support the previous finding that the antibody is sensitive and specific for oligomeric/aggregated species. However, it should be considered that the antibody cannot distinguish between oligomers and filaments; thus, the exact species measured in this study are not known, and whether the identity of the aggregated species present, in addition to the quantity, differ between groups requires further study. Furthermore, while the calibrator used was generated from fibrils, the unknown endogenous species should be further studied using differing technologies in future studies.

Almost all (99%) of the α-Syn present in blood is contained in erythrocytes [13]: plasma contains only 0.1% of blood α-syn, peripheral blood mononuclear cells (PBMCs) 0.05%, and platelets 0.2% [13]. Results obtained in plasma or serum α-Syn are quite inconsistent, likely due factors including differences in assays, sample handling, and, importantly, the extent of hemolysis [7–12, 22, 32]. This factor presents a major challenge, as differing hemolysis will result in high, variable levels of erythrocyte α-Syn contaminating the sample, potentially overwhelming the plasma or serum signal. Our study focused on whether erythrocytes, the major source of blood α-Syn, are altered in PD and controls. Because the erythrocytes were isolated and washed during the preparation procedure, contamination from the membrane or contents of cells disrupted during hemolysis are unlikely to play a major role in our assays, thus minimizing this problem that dramatically affects studies of serum or plasma.

### α-Syn in different components of erythrocytes is altered in PD

The current investigation explored the possible involvement of different erythrocytic α-Syn forms in PD patients. Cytosolic pS129 α-Syn levels were significantly higher in PD, but there was no significant difference in cytosolic aggregated or total α-Syn between PD patients and controls and pS129 and total α-Syn were not associated (Additional file 1: Figure S3B). In contrast, erythrocytic membrane total and aggregated α-Syn levels were associated (Additional file 1: Figure S3D) and were both significantly higher in PD patients. It should also be emphasized that the concentrations of aggregated and pS129 α-Syn were normalized to corresponding total α-Syn levels, indicating that their increases in PD are most likely not due to alterations of total (including monomeric or unphosphorylated) α-Syn levels in the same subcellular compartment. This observation certainly raises the possibility that the increased plasma or serum α-Syn oligomers are derived from erythrocytes, and erythrocytic pS129 could be a major source of plasma pS129.

The formation of Lewy bodies (LBs) is related to the misfolding, oligomerization and aggregation of α-Syn [40]. PD patients had higher levels of α-Syn oligomers in CSF [2, 6, 41] and plasma [21], although the source(s) of these oligomers is unknown. Recent studies have shown increased dimeric α-Syn in erythrocyte membranes [42], and elevated oligomeric/total α-Syn ratio in erythrocytes [30], in PD vs control patients. Further, a few studies have observed differences in post-translationally modified α-Syn forms in erythrocytes between PD patients and healthy controls [43, 44]. However, most of these studies, including our pilot study [29], tested small sample cohorts with less robust immunoassays. When assessing aggregated α-Syn in whole erythrocytes, unfortunately, our previously reported performance for PD diagnosis was largely not confirmed in the current study with a much larger, independent cohort and more robust ECL assays. Additionally, no correlation between erythrocyte α-Syn and disease duration, age, or motor scale score in PD patients has been evidenced [29], and most of these studies examined α-Syn in erythrocyte lysates, potentially missing differences in the sorting of α-Syn by fraction, or sub-populations of the protein, thus highlighting the need for further studies.

In the present study, we found that aggregated α-Syn in the membrane, but not the cytosolic, fraction was significantly higher in PD than in controls. This could be at least partially explained by altered lipid membrane composition in PD [45], which may affect α-Syn and membrane interactions as well as α-Syn aggregation [46]. On the other hand, remarkable morphological disorder of erythrocytes, exhibiting membrane spikes and eryptosis (programmed red cell death), has been reported in PD [18]. Given that α-Syn could progressively aggregate unevenly at the surface of the membrane, and α-Syn oligomers/aggregates could disturb the normal biological membranes, it is possible that increased membrane oligomeric α-Syn contributes to the morphological abnormalities of erythrocytes seen in PD patients [18]. Further support for this argument can be found in studies showing that α-Syn progressively aggregated unevenly at the surface of membrane, and that both lipid and membrane proteins were incorporated in the aggregates [47].

A caveat is that the dopaminergic medication utilized by all PD patients included in this study may be a potential confounding factor, as dopamine is reported to induce α-Syn oligomerization [48]. Whether this factor affects the aggregated α-Syn measurements needs to be further investigated.

α-Syn in Lewy bodies from PD patients is hyperphosphorylated at S129 [49–52], which may contribute to PD pathogenesis, as α-Syn becomes more susceptible to aggregation [23, 24, 50] and more toxic [23–25] when it is converted to pS129. α-Syn phosphorylation may also occur after LB formation [53], and pS129 accumulation in the brain could represent a late event in disease progression [51, 54]. It is thus plausible that the aberrant phosphorylation of α-Syn may promote LB clearance or degradation [53]. Nonetheless, cross-sectional studies in brain tissue and CSF indicate increased phosphorylated α-Syn in PD [22, 50, 55, 56], promoting further investigation of pS129 as a PD biomarker. In the present study, we found that pS129 significantly increased in erythrocytes of PD patients, suggesting that aberrant phosphorylation of α-Syn may also occur in peripheral blood (erythrocytes), consistent with previous findings in plasma [21]. Whether this change in blood occurs during the early or late stages of the disease and whether the peripheral α-Syn changes could contribute to PD development and progression in the brain should be further investigated (see more discussion in section 4.4 below). Nonetheless, our current data suggests that peripheral erythrocytic pS129 could be a novel biomarker for PD diagnosis. Additionally, its correlation with MoCA in PD patients might suggest a relationship between erythrocytic pS129 and PD severity (cognition) to be further investigated.

Notably, the pS129 to total α-Syn ratio in erythrocytic fractions was about 1%. In contrast, the same ratio in CSF was reported to be 15–25% in previous studies [33, 41, 55, 57]. Although these ratios might not be directly comparable, as different pS129 and total α-Syn immunoassays were used in the studies, it is possible that the phosphorylation of α-Syn is pathologically more relevant in CSF than in erythrocytes. That said, because biomarkers in peripheral blood are needed, changes in pS129 in erythrocytes or other blood fractions, likely when combined with other changes such as those observed in the current study, could still be clinical useful.

### Erythrocytic α-Syn for PD diagnosis and severity

We discovered that erythrocytic membrane aggregated α-Syn and cytosolic pS129 are potential biomarkers for PD diagnosis, particularly when coupled with age, where they achieved a sensitivity and specificity comparable to those based on CSF α-Syn values [2, 32], but using a sample source that is both more accessible and less sensitive to hemolysis. They could potentially be further improved by adding other factors, such as erythrocyte Aβ and tau [58]. Whether changes in erythrocytic α-Syn species can be reliably and consistently observed at early or even pre-clinical disease stages, i.e., whether they could be used as early or pre-clinical PD biomarkers, needs to be further studied.

To date, it remains difficult to assess PD progression objectively. Correlation of CSF or peripheral α-Syn with PD severity and/or PD progression has been inconsistent [2, 7, 10–12, 29, 32]., but an association of CSF α-Syn with non-motor symptoms or cognitive decline in PD has been suggested [59–61]. In this study, erythrocytic pS129 correlated modestly with the severity of PD cognitive symptoms, though whether this finding is significant will depend on independent validation, particularly in longitudinally collected samples.

### α-Syn peripheral and central transport and the implications of altered peripheral erythrocytic α-Syn in PD

Earlier observations of aggregated α-Syn in neurons grafted into brains of PD patients suggested cell-to-cell transfer of α-Syn in a possible prion-like fashion [62]. Other studies have shown that non-fibrillar (monomeric or oligomeric) α-Syn can be secreted into the cell medium via cell-derived extracellular vesicles (EVs) such as exosomes [63]. Our recent study found that not only could α-Syn-containing EVs derived from cultured human erythrocytes pass through the blood-brain barrier, but erythrocyte EVs obtained from the blood of PD patients induced a greater pro-inflammatory response in microglia than did those from control subjects [64]. The hypothesis of peripheral-to-CNS transport of α-Syn is surely in line with observations demonstrating: (1) that EVs or exosomes are an important route for transporting α-Syn species from the periphery to the CNS [63, 64]; and (2) erythrocytes play an important role in the peripheral aspects of PD onset [13, 18, 29, 64]. Whether increased erythrocytic and plasma/serum α-Syn observed in the current and previous studies, free or contained in exosomes, could be transported to the CNS, contributing to CNS pathology, remains to be investigated. If such transfer occurs, this study is in line with the idea that peripheral changes in PD could influence the disease development and progression in the brain.

## Conclusions

In summary, this study has demonstrated the usefulness of newly developed ECL assays in assessing total, aggregated and pS129 α-Syn contained in human erythrocytes. Although further independent validation is needed, this investigation suggested that erythrocytic α-Syn species are potential peripheral biomarkers for PD diagnosis, with sensitivity and specificity from an integrated model comparable to what can be achieved by CSF α-Syn. More interestingly, the observed alterations in erythrocytic α-Syn levels in PD, together with other existing evidence, supported that such peripheral changes could influence the disease development and progression in the brain.

## Additional file

Additional file 1: **Figure S1.** Characterization of erythrocyte membrane and cytoplasmic component properties. **Figure S2.** The receiver operating characteristic curve for agrregated α-Syn in whole erythrocytes. **Figure S3.** Correlations between α-Syn species within each subcellular compartment. (DOCX 4486 kb)

## Abbreviations

BBB: Blood-brain barrier
CSF: Cerebrospinal fluid
ECL: Electrochemiluminescence
MoCA: Montreal Cognitive Assessment
PD: Parkinson’s disease
PTM: Post-translationally modified
UPDRS: Unified Parkinson disease rating scale
α-Syn: α-synuclein
