# Supplementary material for: Erythrocytic α-Synuclein as a potential biomarker for Parkinson’s disease
Source: Transl Neurodegener. 2019 May 15;8:15. doi: 10.1186/s40035-019-0155-y (PMC6521422; doi:10.1186/s40035-019-0155-y)
Supplement: Supplementary file 1 — Figure S1. Characterization of erythrocyte membrane and cytoplasmic component properties. Figure S2. The receiver operating characteristic curve for agrregated α-Syn in whole erythrocytes. Figure S3. Correlations between α-Syn species within each subcellular compartment. (DOCX 4486 kb) [file 40035_2019_155_MOESM1_ESM.docx]

**Erythrocytic α-Synuclein as a Potential Biomarker for Parkinson’s Disease**

**Supplemental Figures**


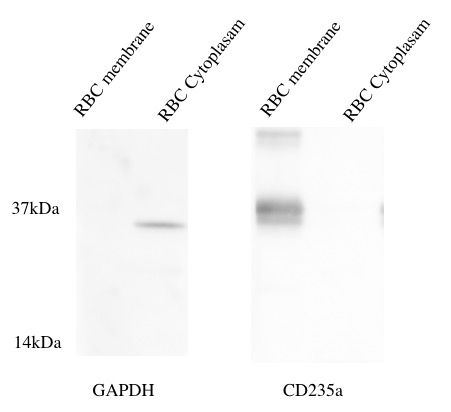


**Supplemental Figure 1.** **Characterization of erythrocyte membrane and cytoplasmic component properties.** To determine the quality of the separation of erythrocyte membrane and cytoplasmic components, western blots were performed using antibodies against proteins that are specific to membrane and cytoplasmic fractions. Glycophorin A (CD235a) is a sialoglycoprotein present on erythrocyte membrane and glyceraldehyde-3-phosphate dehydrogenase (GAPDH) is in erythrocytic cytoplasm. CD235 was expressed only on the erythrocytic membrane and GAPDH was expressed only on the erythrocytic cytoplasm, suggesting that the erythrocyte membrane and cytoplasm were properly separated.


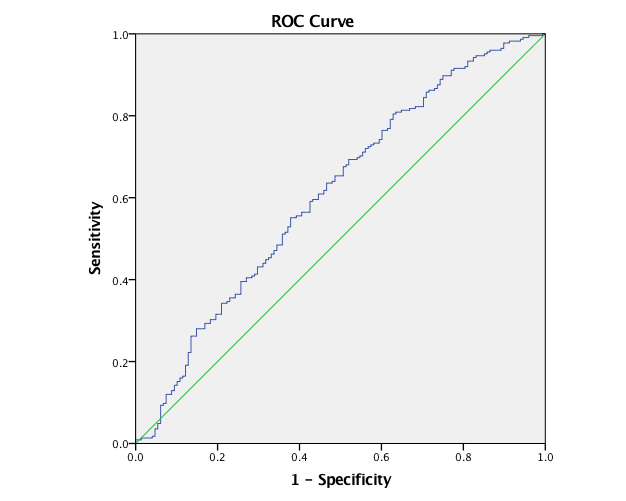


**Supplemental Figure 2. The receiver operating characteristic curve for agrregated α-Syn in whole erythrocytes.** Aggregated α-Syn and total protein concentrations in whole erythrocytes were measured and the α-Syn aggregate/erythrocyte total protein ratio was evaluated for PD diagnosis in this study (AUC=0.6).


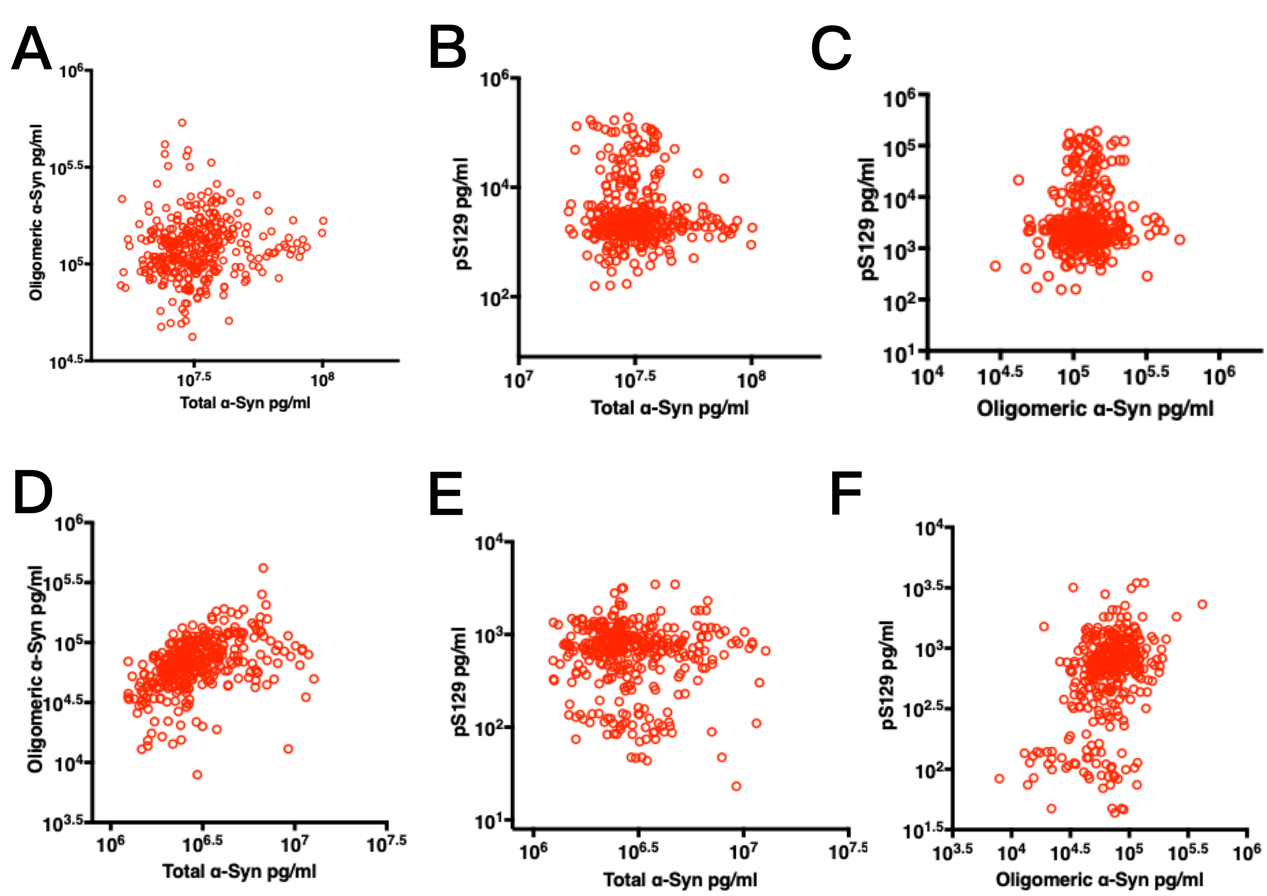


**Supplemental Figure 3. Correlations between α-Syn species within each subcellular compartment.** (A) Correlation between total α-Syn and aggregated α-Syn in erythrocyte cytosol (*p*=0.002, ρ^2^=0.028). (B) Correlation between total α-Syn and pS129 in erythrocyte cytosol (*p*=0.85). (C) Correlation between aggregated α-Syn and pS129 in erythrocyte cytosol (*p*=0.26). (D) Correlation between total α-Syn and aggregated α-Syn in erythrocyte membrane (*p*<0.001, ρ^2^=0.25). (E) Correlation between total α-Syn and pS129 in erythrocyte membrane (*p*=0.30). (F) Correlation between aggregated α-Syn and pS129 in erythrocyte membrane (*p*<0.001, ρ^2^=0.11). All correlations are evaluated using Spearman’s Spearman's rank correlation coefficient (ρ).
